# Customizable Bayesian adaptive testing with Python – The *adaptivetesting* package

**DOI:** 10.3758/s13428-026-03079-w

**Published:** 2026-07-24

**Authors:** Jonas Engicht, R. Maximilian Bee, Tobias Koch

**Affiliations:** Institute of Psychology, Friedrich-Schiller University Jena, Am Steiger 3, Haus 1, 07743 Jena, Germany

**Keywords:** Computerized adaptive testing, Item response theory, Python package, Bayesian statistics

## Abstract

This paper introduces an open-source Python package for simplified, customizable computerized adaptive testing (CAT) using Bayesian methods for ability estimation. It addresses the lack of sophisticated packages for CAT in the Python programming language. Moreover, it bridges the gap between the construction and simulation of adaptive tests and their practical application by providing a dedicated API for integration with experiment software. Thereby, it eliminates the need for major code rewrites when transitioning from simulated to real-world adaptive testing. By leveraging Python’s object-oriented programming approach, such as abstract classes, protocols, and inheritance, the package allows for easy extension and customization of its functionality. For example, Bayesian estimators can be modified to incorporate custom priors. This paper outlines the relevance and practical use of the adaptivetesting package through a walkthrough example. The package is fully documented, and its source code is published on GitHub. It is also available on the Python Package Index (PyPi) and conda-forge thus it can easily be installed using Python’s package manager pip or conda. Leveraging R’s reticulate package, adaptivetesting can also be accessed from within RStudio.

## Introduction

Computerized adaptive testing (CAT) offers a powerful and economic alternative to traditional fixed-form assessments by dynamically selecting only the most diagnostically informative items for each respondent. By tailoring item presentation in this way, CAT can substantially reduce test length and participants’ burden while retaining measurement precision comparable to that of full-length assessments (Wainer et al., 2000). This makes CAT a highly practical approach for both researchers and practitioners, particularly when time and resources are limited.

To utilize CAT, certain prerequisites must be met. First, an item pool is required that fits an item response theory (IRT) model. Moreover, appropriate algorithms must be selected for estimating the participant’s latent ability and for selecting new informative items. In addition to traditional frequentist methods, CAT can also be used with Bayesian methods. On the one hand, ability estimation and item selection can be performed using Bayesian methods. Additionally, and therefore also called fully Bayesian CAT, uncertainty in item parameter estimation can also be considered using Bayesian methods (Fink et al., 2025; Niu & Choi, 2022; Ren et al., 2020; Van Der Linden & Ren, 2020).

There are several software solutions and packages to implement CAT. For the R programming language, the catR package (Magis & Raîche, 2012; Magis & Barrada, 2017) offers sophisticated functionality for researchers working with CAT. However, despite its wide range of features, the package has certain important limitations. First, it is primarily designed for simulating adaptive tests and does not offer a user-friendly interface for administering tests in real applications. Second, with regard to Bayesian ability estimation, it supports only three types of priors: uninformative, normal, and Jeffreys’ priors. This represents a notable limitation, as there are use cases where alternative prior distributions may be more important. Moreover, the package is not easily extensible. For example, incorporating additional prior distributions would require substantial modifications and refinements of the original package.

For the Python programming language, several packages for CAT already exist, such as catSim (Meneghetti & Aquino, 2018) and EduCAT (Liu et al., 2024). However, these packages lack important functionality. For instance, catSim is limited to the simulation of CAT, while EduCAT adopts a machine learning approach and does not allow customizations of the already implemented procedures. Moreover, neither package supports Bayesian ability estimation.

Therefore, none of these packages represents a viable option for researchers and practitioners who wish to employ Bayesian methods for their adaptive tests while also requiring flexible options for test modeling and construction.

This paper introduces adaptivetesting – a Python package that simplifies the development and deployment of adaptive tests. It provides researchers with full control over the test flow while offering a common and standardized programming interface that leverages Python’s object-oriented programming language features. The package is specifically designed to support Bayesian methods for ability estimation, directly addressing the limitations of existing, established packages. Furthermore, adaptivetesting aims to bridge the gap between the construction, simulation, and practical application of adaptive tests by providing a dedicated interface compatible with testing software, such as PsychoPy (Peirce et al., 2019). This integration reduces the need for major code rewrites and minimizes the risk of errors that can arise when re-implementing a test in another language or software.

During the development of the package, the R package catR was used as a benchmark to ensure that all calculations and functions have been correctly implemented and produce equivalent results within a small margin. Using methods and procedures implemented in both packages, the test outcomes are consistent, with only minor numerical differences attributable to differences in implementation.

The source code of the adaptivetesting package is fully documented and publicly available on GitHub^1^ under the Mozilla Public License Version 2.0. It is therefore open for adaptation and contributions from the open-source community. The package is also available in the Python Package Index (PyPi) and conda-forge (Conda-Forge Community, 2015). It supports Python versions ≥3.12. The functionality of the package can also be accessed via RStudio (Posit team, 2025), an interface more familiar to R users, by leveraging the reticulate R package (Ushey et al., 2023).

## Overview

This paper is organized as follows. First, an introduction to item response theory (IRT) and computerized adaptive testing (CAT) is provided. Second, the package’s functionality and technical aspects related to the implementation of CAT are presented. Third, the package is illustrated through a practical example involving the use of an IRT-based item pool from a report measure assessing children’s expressive vocabulary. The accompanying software tutorial demonstrates the full flexibility of the package and showcases its broad range of possible applications – from test development to practical deployment.

## Item response theory

In psychological research, questionnaires and tests often consist of dichotomous response variables, such as true/false or yes/no questions, or polytomous response variables, such as unordered categories. Item response theory (IRT) is a formal measurement theory that can be applied to analyze data with dichotomous or polytomous categorical response variables.

**Fig. 1:**
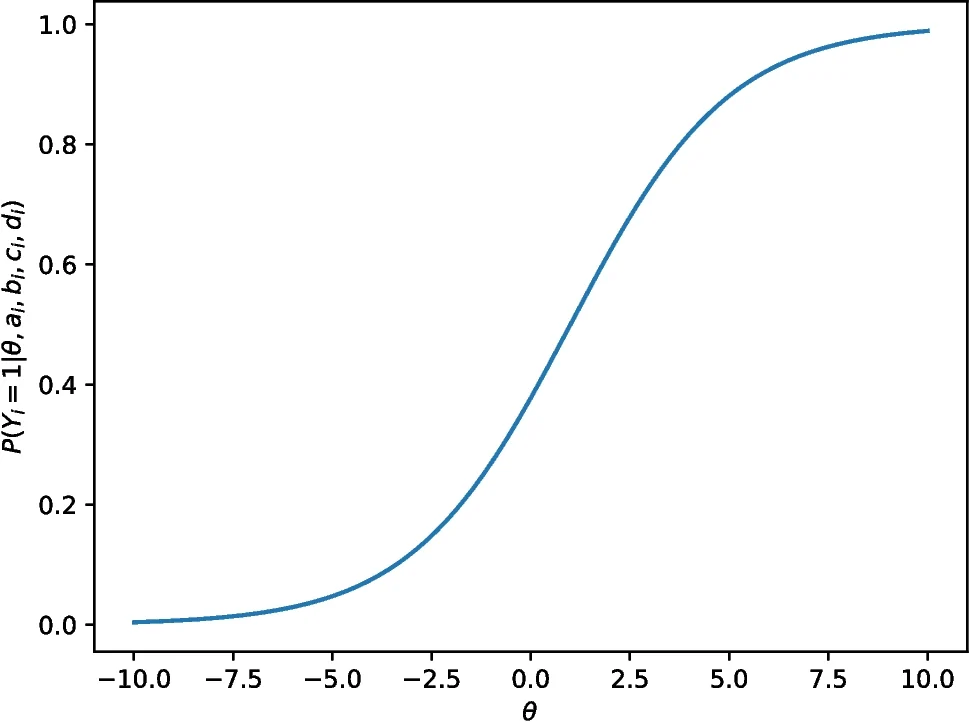
Item characteristic curve (ICC) for an example item *i*

For dichotomous response variables, consider an item *i* in a set of items *N* and the participant’s latent ability $\theta \in \mathbb {R}$ with a response variable $Y_i \in \{0, 1\}$. In this context, IRT can be used to model the probability of a correct answer $P(Y_i=1 | \theta )$ (Hambleton et al., 1995). The basic idea of IRT is that the higher the participant’s latent ability, the more likely they are to answer an item correctly. Hence, the probability of a correct answer increases. This relationship can be visualized using an item characteristic curve (ICC), as shown in Fig. 1 (Hambleton et al., 1995).

The library matplotlib (Hunter, 2007) was employed for the creation of this graphic.

A rather unrestrictive IRT model is the four-parameter logistic (4PL) model proposed by Barton et al. (1981). It assumes that the probability $P(Y_i=1)$ is a function of the participant’s ability θ and four item-specific parameters $a_i$ (discrimination), $b_i$ (difficulty), $c_i$ (pseudo-guessing), and $d_i$ (inattention), resulting in the following equation:

1 $$\begin{aligned} P(Y_i=1 | \theta ,a_i,b_i,c_i,d_i)=c_i+(d_i-c_i) \frac{\exp ^{a_i(\theta -b_i)}}{1+\exp ^{a_i(\theta -b_i)}} \end{aligned}$$

By imposing constraints on the item parameters, several more restrictive IRT models can be derived, such as the three-parameter logistic (3PL) model, two-parameter logistic (2PL) model (Birnbaum, 1968), and the Rasch model (Rasch, 1980). For more information, see, for example, Magis and Raîche (2012).

All of these IRT models share two common assumptions: unidimensionality and local independence (Hambleton et al., 1995). Unidimensionality means that one latent ability dimension is measured by all of the test items. Local independence implies that the joint probability of item responses $Y_1, Y_2, ..., Y_n$ is equivalent to the product of the item probabilities $P(Y_i|\theta )$ (Lord & Novick, 1968), so that

2 $$\begin{aligned} P(Y_1, Y_2, ..., Y_n | \theta ) = \prod _{i=1}^{n} P(Y_i | \theta ) \end{aligned}$$

If the assumption of local independence holds, the participants’ responses are statistically independent given a fixed latent ability level. In other words, once the latent ability is accounted for, item responses are conditionally independent, indicating that the ability explains all systematic relationships among the test items (Hambleton & Swaminathan, 2010).

IRT models can also be applied to polytomous response variables with ordered categories. Two selected IRT models, which can be used in this case, are the Graded Response Model (GRM) (Samejima, 1969) and the Generalized Partial Credit Model (GPCM) (Muraki, 1992). In these models, the responses to items, denoted as *k*, can be categorized into $g_i + 1$ possible outcomes, meaning *k* can take any value from the set $\{0, 1, ..., g_i\}$. Furthermore, each item is characterized by two statistical parameters: $a_i$ and the category threshold parameters $b_i$ (i.e., $b_i = (b_{i1}, b_{i2}, ...)$). With the GRM, the appropriate item parameters and latent ability, one can calculate the probability of a response $Y_i$ in category *k* or a higher category, so that

3 $$\begin{aligned} P(Y_i \ge k | \theta , a_i, b_{i}) = \frac{\exp ^{a_i(\theta - b_{ik})}}{1 + \exp ^{a_i(\theta - b_{ik})}} \end{aligned}$$

Because the GRM is a difference model (Thissen & Steinberg, 1986), the probability of a response $Y_i$ in category *k* can be calculated from

4 $$\begin{aligned} P(Y_i = k | \theta , a_i, b_{i}) = P(Y_i \ge k | \theta , a_i, b_{i}) - P(Y_i \ge k + 1| \theta , a_i, b_{i}) \end{aligned}$$

The GPCM is a divide-by-total model (Thissen & Steinberg, 1986) so that

5 $$\begin{aligned} \frac{P(Y_i = k | \theta , a_i, b_i)}{P(Y_i = k | \theta , a_i, b_i) + P(Y_i = k-1 | \theta , a_i, b_i)} = \frac{\exp ^{a_i (\theta - b_{ik})}}{1 +\exp ^{a_i (\theta - b_{ik})}} \end{aligned}$$

Note that in both models, the number of categories can differ for every item. The GPCM can be restricted to the Partial Credit Model and its variants, aligning more closely with the tradition of the Rasch model. It offers greater flexibility in cases of unordered or asymmetrical thresholds. In contrast, the GRM is often advantageous when response categories are best understood as ordered cumulative thresholds, which can make it especially natural for Likert-type items and easier to interpret in terms of the probability of endorsing increasingly higher categories.

## Computerized adaptive testing

Using IRT as a psychometric foundation enables researchers to implement computerized adaptive testing (CAT). In this paradigm, only the most informative items are administered to each participant, rather than the full-length test (Magis & Raîche, 2012). This approach can lead to shorter tests while retaining equivalent measurement precision equivalent to that of their full-length counterparts (Wainer et al., 2000).

CAT is particularly advantageous for both researchers and participants when there are limited resources or in high-stakes testing situations (e.g., intelligence testing, SAT).

**Fig. 2:**
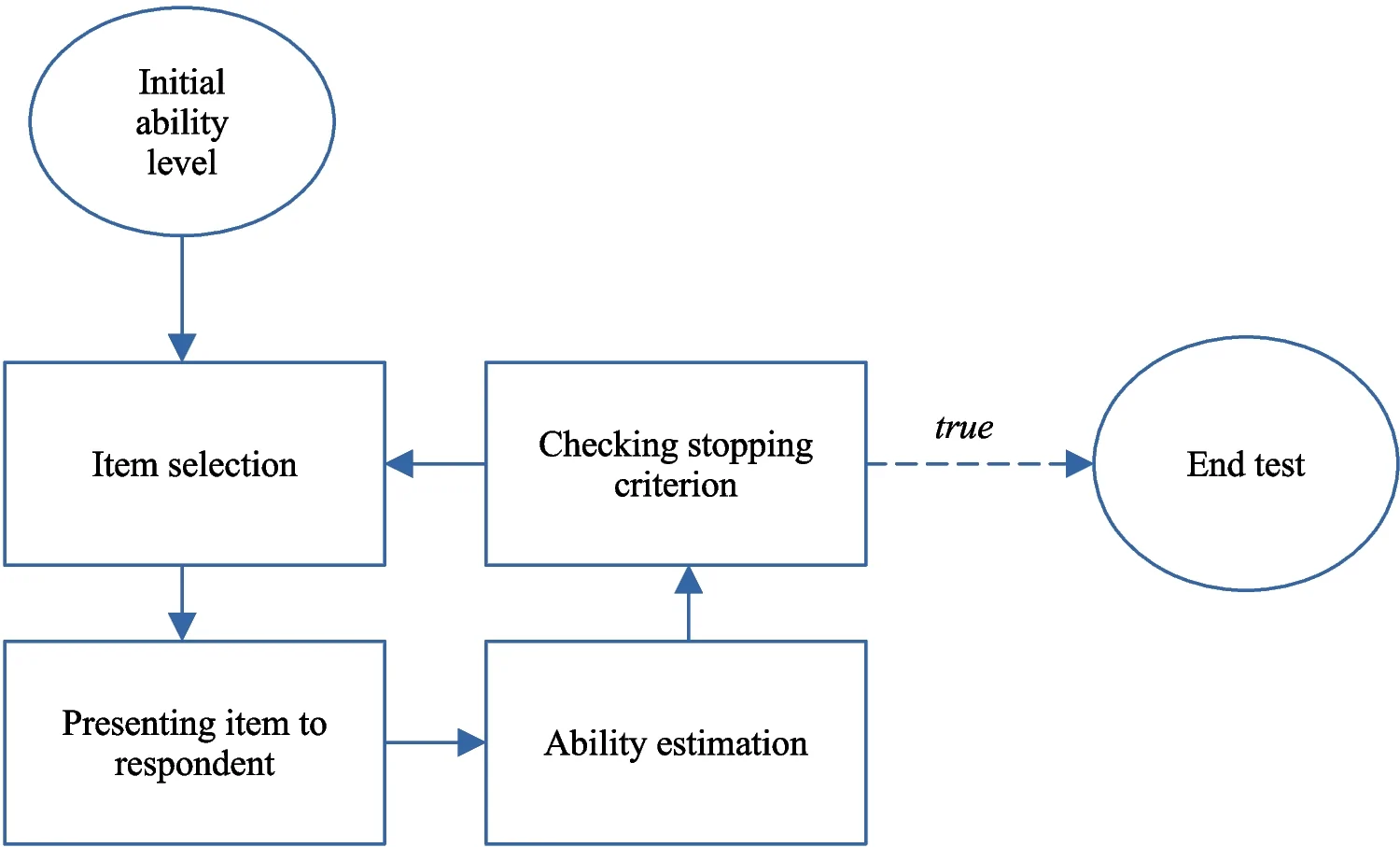
Schematic overview of computerized adaptive testing (CAT)

Following Magis and Raîche (2012), CAT usually begins by assuming an initial estimate of the participant’s latent ability. Based on this estimate, a highly informative item is selected and presented to the respondent. Once the response is recorded, the latent ability is re-estimated and, if necessary, updated. Next, a stopping criterion (e.g., predefined precision value) is evaluated. If the criterion is met, the test concludes. Otherwise, a new informative item is selected from the item pool (Magis & Raîche, 2012). Figure 2 graphically illustrates this process.

According to Frey (2023), an adaptive test can be broken down into various components, also called building blocks, which represent design options for testing procedures. One fundamental aspect of an adaptive test is the item pool. It represents the complete set of items that can potentially be used during a testing procedure. The item parameters are estimated in a calibration study before the application in CAT. However, with online calibration, for example, the estimation of parameters for new items can also be performed while administering previously calibrated ones (Wainer & Mislevy, 2000).

An adaptive test can also be started in various ways.Frey (2023) points out that at the beginning, no previous answers are available, and therefore no informative item selection based on prior response behavior can take place. However, if available, information from previous tests or similar sources can be used. Otherwise, Frey (2023) suggests choosing a random item or so-called “icebreaker” items. Furthermore, there are certainly alternative methods to begin a test. For example, Bohn et al. (2025) chooses an item of average difficulty to start with.

To estimate the latent ability parameters of test-takers, researchers can choose from multiple estimation methods. This allows for the use of classical maximum likelihood estimators, among others. However, Bayesian methods such as expected a priori (EAP) or Bayes modal (BM) may also be employed.

As previously described, CAT aims to select highly informative items. However, there are also other criteria for item selection that can be used, such as Urry’s rule (Urry, 1970) or the maximum expected information criterion (Van Der Linden, 1998). A comprehensive exploration of this topic is beyond the scope of this discussion. For further details, refer to Van Der Linden (2005) and Choi and Swartz (2009). Items can also be grouped into testlets, as is done, for example, in PISA and PIRLS, or into different stages which are then assembled in advance (multistage testing) (Frey, 2023).

Aside from selecting items that are as informative and therefore statistically relevant as possible, non-statistical constraints can also be implemented in an adaptive test (Frey, 2023). This so-called constraint management can be subdivided into exposure control and content balancing. Exposure control influences how often items are presented across tests, whereas content balancing influences item selection within test-runs and is often used to control the type of items presented to participants (Frey, 2023). A wide range of methods is available for implementing constraint management. Possible methods for exposure control would include, among others, randomesque item selection (Kingsbury & Zara, 1989, 1991) and the Maximum Priority Index method (Cheng & Chang, 2009). Content balancing can, for example, be performed with the Weighted Penalty Model (Shin et al., 2009) or also with the Maximum Priority Index method (Cheng & Chang, 2009).

Several stopping rules may be applied to an adaptive test, which should be selected in accordance with the diagnostic goal of the test. For instance, a test can stop if (a) the item pool is empty, (b) the maximum test length has been reached, (c) the standard error of the ability estimate has reached a critical threshold, (d) the ability estimate has reached a threshold, or (e) the maximum test duration has been reached (Frey, 2023; Linacre, 2000).

**Table 1:** Overview of the features implemented in adaptivetesting

**Ability estimators**	
Maximum likelihood	$\arg \max \, L(Y=1|\theta )$
Bayes modal	$\arg \max \frac{L(\theta )P_\text {prior}(\theta )}{\int _{-\infty }^\infty L(\tilde{\theta })P_\text {prior}(\tilde{\theta }) d{\tilde{\theta }}}$
Expected a posteriori	$\frac{\int _{-\infty }^{\infty } \theta L(\theta ) P_\text {prior}(\theta )\, d\theta }{\int _{-\infty }^{\infty } L(\theta )P_\text {prior}(\theta )\, d\theta }$
**Item selection**	
MFI	$\arg \max \, I_{\tilde{i}}(\hat{\theta })$
**Exposure control**	
Randomesque	random draw from the *n* most informative items
Maximum Priority Index	$PI_i = I_i \prod _{k=1}^K (w_k f_k)^{c_{ik}}$
**Content balancing**	
Maximum Priority Index	$PI_i = I_i \prod _{k=1}^K (w_k f_k)^{c_{ik}}$
Weighted Penalty Model	$F_i = w'F_i' + w'' F_i''$
**Stopping criterion**	
Standard error	$\le \frac{1}{\sqrt{I(\theta )}}$
Test length	$\#\, \text {of items}$

Apart from modifications in these standard building blocks of CAT, tests can be modified to control for item position effects, which often occur in international large-scale assessments and may lead to biased results (Frey , Fink, 2025). There are different methods to respond to those effects. For instance, Ma and Harris (2025) proposes (a) adjusting item parameter estimates at an item level to consider position effects, (b) controlling effects through a pretest, or (c) controlling for item administration positions at the item pool level. Moreover, if adaptive tests are applied over a longer period of time, item parameter drift (IPD) can occur – that is, a special form of differential item functioning leading to differential change of item parameters over time (Bock et al., 1988; Goldstein, 1983). Notably, IPD can be handled by using time-dependent IRT models (Bock et al., 1988). However, these topics exceed the scope of this paper and therefore will not be covered further.

It can be concluded from these statements that there is a high degree of flexibility in many areas of CAT and that many design decisions have to be made during test development. Additionally, implementing CAT often involves considerable effort, as CAT scenarios are typically extensively simulated prior to their practical application to ensure their suitability for the intended context and scope (Frey, 2020). Moreover, in many research settings, custom-built CAT procedures may be necessary to meet specific measurement needs.

## Features

### Ability estimation

Table 1 provides an overview of the features implemented in the package. The package includes three different ability estimators: traditional maximum likelihood estimation (ML), Bayes modal (BM), and expected a posteriori (EAP). ML uses the ability value θ as estimate $\hat{\theta }$ that maximizes the likelihood function L(θ) (Lord, 2012), such that

6 $$\begin{aligned} \hat{\theta }_{ML}&= \arg \max \, L(Y=1|\theta )\end{aligned}$$

7 $$\begin{aligned} \hat{\theta }_{ML}&= \arg \max \ P(Y_1, Y_2, ..., Y_n | \theta ) \end{aligned}$$

The BM estimator, also called maximum a posteriori, maximizes the posterior distribution (Birnbaum, 1969), so that

8 $$\begin{aligned} \hat{\theta }_{\text {BM}} = \arg \max \frac{L(\theta )P_\text {prior}(\theta )}{\int _{-\infty }^\infty L(\tilde{\theta })P_\text {prior}(\tilde{\theta }) d{\tilde{\theta }}} \end{aligned}$$

with the probability density function of the prior $P_\text {prior}(\theta )$. If a uniform prior is used, the BM estimator is equal to the ML estimator. The package adaptivetesting permits researchers to use any continuous one-dimensional probability density function as a prior distribution.

The package implements three different prior distributions that can be used for Bayesian ability estimations: a normal distribution, a skew normal distribution, and an empirical prior. The empirical prior is constructed from an empirical sample using a kernel density estimate to provide a nonparametric prior estimated from observed data. Additionally, distributions already implemented in the scipy package (Mckinney, 2010) can readily be used. However, custom priors may also be specified by defining their probability density functions. These functions, however, must be numerically differentiable and integrable to ensure proper computational handling.

The EAP estimator (Bock & Mislevy, 1982) uses the mean of the posterior distribution.

9 $$\begin{aligned} \hat{\theta }_\text {EAP} = \frac{\int _{-\infty }^{\infty } \theta L(\theta ) P_\text {prior}(\theta )\, d\theta }{\int _{-\infty }^{\infty } L(\theta ) P_\text {prior}(\theta )\, d\theta } \end{aligned}$$

Depending on the estimator used, a standard error of the person’s ability estimate is calculated. The standard error can be interpreted as an indicator of the reliability of the ability estimate. For ML and BM, the standard error depends on the test information function I(θ), which itself is derived from the item information function (IIF) $I_i(\theta )$, and, if applicable, the information is weighted by the prior distribution $I_\text {Prior}(\theta )$.

Following Magis and Raîche (2012), the standard error of the Bayes modal estimator $\hat{\theta }_\text {BM}$ is defined as

10 $$\begin{aligned} se_{\hat{\theta }_\text {BM}} = \frac{1}{\sqrt{I(\hat{\theta }_\text {BM})}}, \end{aligned}$$

where I(θ) is the test information function. The information function of a single test item *i* is defined as the Fisher information of that item (Johnson, 2018). In case of the 4PL model (see Eq. 1), the Fisher information takes the following form (Magis & Raîche, 2012):

11 $$\begin{aligned} I_i(\theta ) = \frac{(\frac{\text {d}}{\text {d}\theta } P_i(\theta ))^2}{P_i(\theta ) Q_i(\theta )}, \end{aligned}$$

where P(θ) is the probability of a correct response (success) and $Q_i(\theta )$ is the probability of an incorrect response (failure or counter event). For polytomous models, the item information can be calculated from the category probability $P_{ik} (\theta )$ and its first derivative $\frac{\text {d}}{\text {d}\theta } P_{ik}(\theta )$ for all *n* response categories (Dodd et al., 1995; Embretson, 2009; Samejima, 1969):

12 $$\begin{aligned} I_i(\theta ) = \sum _{k=0}^{n} \frac{(\frac{\text {d}}{\text {d}\theta } P_{ik}(\theta ))^2}{P_{ik}(\theta )} \end{aligned}$$

The sum of each single item information represents the informational value of the whole item pool (Johnson, 2018). However, when using a Bayesian estimator, the prior distribution has to be considered.

The Fisher information of the prior can be calculated from its probability density function $P_\text {prior}(\theta )$. For that purpose, the score function S(θ) of the prior is used, which is defined as follows (Casella & Berger, 2002)

13 $$\begin{aligned} S(\theta ) = \frac{\text {d}}{\text {d}\theta } \ln P_\text {prior}\,(\theta ) \end{aligned}$$

Hence, the Fisher information is given by (Casella & Berger, 2002)

14 $$\begin{aligned} I_\text {prior} = E[S(\theta )^2] \end{aligned}$$

and can be numerically calculated using

15 $$\begin{aligned} I_\text {prior} = \int _{-\infty }^{\infty } S(\theta )^2 \cdot P_\text {prior}(\theta ) d\theta \end{aligned}$$

Because item information functions are additive under the assumption of local independence and assuming the same holds for the prior information, the test information function for Bayes modal estimators, which incorporates the prior, is given by Johnson (2018)

16 $$\begin{aligned} I(\theta ) = \sum _{i = 1}^{n} I_i(\theta ) + I_\text {prior}(\theta ) \end{aligned}$$

For the EAP estimator, the standard error is relatively straightforward to compute, as it is defined as the standard deviation of the posterior distribution, $P_\text {posterior}(\theta )$, which can be calculated using (Bock & Mislevy, 1982)

17 $$\begin{aligned} se_{\hat{\theta }_\text {EAP}}&= \sqrt{Var_\text {posterior}(\theta )}\end{aligned}$$

18 $$\begin{aligned} se_{\hat{\theta }_\text {EAP}}&= \sqrt{\frac{\int _{-\infty }^\infty (\theta - \hat{\theta })^2 P_\text {prior}(\theta ) L(\theta ) d\theta }{\int _{-\infty }^\infty P_\text {prior}(\theta )L(\theta ) d\theta }} \end{aligned}$$

### Item selection

To select new items, adaptivetesting utilizes the maximum Fisher information (MFI) criterion (Lord, 1977). Thereby, the item *i* is selected that maximizes the information function (Lord, 1977; Magis & Raîche, 2012)

19 $$\begin{aligned} i = \arg \max \, I_{\tilde{i}}(\hat{\theta }) \end{aligned}$$

### Constraint management

#### Content balancing

The Weighted Penalty Model (WPM) (Shin et al., 2009) can be used to balance content properties and non-statistical constraints. Thereby, a tradeoff is made between the item information and the importance of constraints. A constraint *k* is defined by (a) an upper proportion bound $Upper_k$ of the item proportion associated with constraint *k*, (b) an lower proportion bound $Lower_j$ of the item proportion associated with constraint *k*, (c) the midpoint between the upper and lower bounds, and (d) the prevalence $Prevalence_k$, which is the proportion of items in the item pool relevant for constraint *k*.

For every item, the total content penalty value is calculated, taking into account all relevant constraints for an item. The WPM also considers the item information, given the currently estimated ability, and calculates an item penalty value. Both the (standardized) content penalty value $$F_i'$$ and information penalty value $$F_i''$$ are weighted with the content constraint weight $w^{\prime }$ and item information weight $w^{\prime \prime }$. Thus, the final weighted penalty value $$F_i$$ is

20 $$\begin{aligned} F_i = w'F_i' + w'' F_i'' \end{aligned}$$

Shin et al. (2009) proposes either setting the weights to fixed values or defining them as functions of the item number in the test.

The WPM then flags the constraints, whether their upper or lower bounds have been reached or not. Additionally, the items are placed in groups depending on their assigned and flagged constraints. These groups are then systematically ordered, and the items within each group are ordered by their weighted penalty value. From the resulting, combined list of items, the package selects the first item.

It should be noted that, of course, the specific rule sets of the WPM are not covered in detail here. For more detailed information on the calculation and item selection procedure of the WPM, see Shin et al. (2009) as this is well beyond the scope of this paper.

Moreover, the Maximum Priority Index method (Cheng & Chang, 2009) is also available for package users. For that, items are organized in groups (content areas) where each group has a corresponding constraint on how many items from this group may be shown during a test. Each item may also be part of multiple content areas. The main idea of the MPI is to change the information value $I_i$ of an item *i* by adding a factor for each relevant constraint *k* that includes the weight of a constraint $w_k$ and its “quota left” $f_k$. A constraint relevancy matrix $\textbf{C}_{I \times K}$ is used to indicate whether a constraint is relevant for a specific item ($c_{ik} = 1$) or not ($c_{ik} = 0$). The “quota left” of a content area is determined by

21 $$\begin{aligned} f_k = \frac{X_k - x_k}{X_k} \end{aligned}$$

with $X_k$ required items from a content area and $x_k$ previously shown items from a content area, while the weight of a constraint $w_k$ is determined deterministically. The final priority index of an item $PI_i$, which is then used for item selection, is calculated as follows

22 $$\begin{aligned} PI_i = I_i \prod _{k=1}^K (w_k f_k)^{c_{ik}} \end{aligned}$$

There are multiple extensions of this method that are currently not supported by the package.

#### Exposure control

For exposure control, the package implements a rather simple method: randomesque item selection. Instead of selecting the single most informative item to be administered next, a random draw is made from the *n* most informative items (Kingsbury & Zara, 1989, 1991). However, the package also allows the use of any arbitrary objective function for item selection.

The Maximum Priority Index method can also be used for exposure control. The “quota left” of each constraint $f_k$ is thereby calculated from the constrained exposure rate $r_k$, the number of previous examinees *N*, and $n_k$ participants who have seen an item from constraint *k*. Thus, $f_k$ is calculated as follows

23 $$\begin{aligned} f_k = \frac{r_k - \frac{n_k}{N}}{r_k} \end{aligned}$$

To apply an exposure rate *r* to all items at once, as it was originally intended by Cheng and Chang (2009), all items have to be assigned to the same constraint.

### Stopping criteria

The package is designed to allow the use of predefined stopping criteria for CAT simulations. These allow tests to be stopped automatically when a specified test length (i.e., a predetermined number of items presented to the participant) or a standard error value (reliability threshold) has been reached. Of course, tests also stop when the item pool is empty.

Furthermore, users can also easily implement functions, for example, in practical test applications, so that tests stop when a latent ability threshold or a maximum test duration has been reached.

### Plots and utility functions

The package also offers features for test diagnostics and easy evaluation of results. On the one hand, functions are included for creating plots. These functions allow to easily plot final ability estimates, ICCs, IFFs, test information functions, and theta estimation traces. Users can also create plots showing the item exposure rate. These functions return matplotlib axis objects, which can then be used to apply further customizations to the plots as needed.

Additionally, several performance evaluation metrics have been implemented in the package, such as bias, residual mean squared error (RMSE), and average absolute deviation (AAD). Given *n* examinees, bias, RMSE, and AAD can be calculated from the final ability estimates $\hat{\theta }_m$ of participant *m* and their corresponding true ability level $\theta _m$, so that

24 $$\begin{aligned} \text {Bias}&= \frac{\sum _m^n (\hat{\theta }_m - \theta _m)}{n}\end{aligned}$$

25 $$\begin{aligned} \text {RMSE}&= \sqrt{\frac{\sum _m^n (\hat{\theta }_m - \theta _m)^2}{n}} \end{aligned}$$

26 $$\begin{aligned} \text {AAD}&= \frac{\sum _m^n |\hat{\theta }_m - \theta _m|}{n} \end{aligned}$$

Moreover, the package, of course, offers functionality to simply parse test results of single participants or whole simulations and data collection samples.

### Feature extensibility

The package is designed to facilitate easy modification and extension of its functionality and features. This flexibility enables researchers to tailor the package to their specific needs while maintaining a consistent and user-friendly interface. Extensions and modifications can range from minor adjustments to the testing procedure to the implementation of entirely new item selection rules, all without requiring substantial changes to the existing, functional code base.

To enable this level of flexibility, the package relies heavily on abstract classes and class inheritance – a common pattern in object-oriented programming. Functionality can then be modified by creating a new class that inherits from the base class or one of its subclasses, with the option to override any relevant function. Additionally, the package uses protocols, which leverage the concept of structural subtyping to define flexible and interchangeable interfaces (The Python Typing Team, 2025).

Although modifications and extensions may not be as straightforward as using the package’s existing functionality, we aim to make this process as accessible as possible by providing highly annotated code and comprehensive documentation, both of which are available in the package’s GitHub repository.

This package design enables researchers to easily adapt the software to their needs while preserving all of its features, without requiring substantial code rewrites. Moreover, as long as the defined interfaces and protocols are correctly implemented, all package functionalities will continue to operate as expected. Nonetheless, it is worth noting that extending and modifying the package requires not only a theoretical understanding of IRT and CAT, but also a significant background in Python and object-oriented programming.

### Package structure

The package follows the standard Python package structure, meaning it is organized into submodules. However, all relevant, user-facing functions are available from the package’s top-level namespace.

The data and models modules provide classes for data management, processing of test results, and defining key structures used throughout the package. Mathematical functions and utilities are defined in the math module. The most important class of the package, the TestAssembler, is included in the implementations module. It offers an intuitive and easy-to-use interface for constructing adaptive tests while allowing for easy modifications. The abstract classes and protocols are specifically designed for extensibility and are located in the dedicated services module, making these definitions easily identifiable and accessible.

Utility functions, for example, for plotting final ability estimates, ICCs, and IIFs, as well as performance metrics, are defined in the utilities submodule. The extra module serves to distinguish the functions clearly from the other package components, which are more closely related to the direct execution of the adaptive tests.

Additionally, the tests module includes unit tests for all key calculations and features in the package. This ensures that code changes do not compromise the package’s critical functionality, which might otherwise go undetected until runtime.

## Application example: Child language research

We illustrate the functionality of the package using empirical data from child language research. The data originate from a study by Bohn et al. (2025), who developed a parent-report instrument for assessing expressive vocabulary in children, known as PREVIC.

In this measure, parents are presented with a list of words and asked to indicate whether their child already produces each word. The PREVIC item pool comprises 89 items and is specifically designed for German-speaking children and parents. Both the test and the item pool are publicly available on GitHub^2^.

We use this item pool to demonstrate both the potential of ada-ptive testing and the versatility of the adaptivetesting package. In an adaptive format, caregivers are presented only with items that are algorithmically selected to efficiently assess the child’s expressive vocabulary. It is important to note, however, that the PREVIC is already implemented as an adaptive test. This highlights the growing adoption of adaptive testing in psychological assessment and child language research, further underscoring the relevance of this example for demonstrating the package’s capabilities.

In the following, we will look at an example of how to simulate adaptive tests using the interface of adaptivetesting. We will then demonstrate how to connect an experiment software to the API to conduct real data collection. We assume a working Python installation (version ≥3.12 or later) and a basic familiarity with the language. A comprehensive introduction to Python lies beyond the scope of this paper.

**Table 2:** Selection of the first five items from the PREVIC item pool and the data provided for each item (Bohn et al., 2025)

word	Difficulty	EstErrorIntercept	Q25Intercept	Q975Intercept
Alternative	2.1851	0.2227	-2.6153	-1.7385
Anzahl	0.2897	0.2209	-0.7178	0.1456
Ausnahme	-1.2650	0.2246	0.8292	1.7073
Bedingung	1.2132	0.2210	-1.6409	-0.7836
Betrieb	1.4860	0.2212	-1.9158	-1.0513

*Note*. The column names in the table represent the names of the columns in the item pool CSV file. The numbers shown are not rounded to depict the referenced data adequately

### CAT simulation

#### Step 1: Package installation and item pool setup

First, we need to install the package adaptivetesting from either PyPi using Python’s package manager pip with

**Figure:**
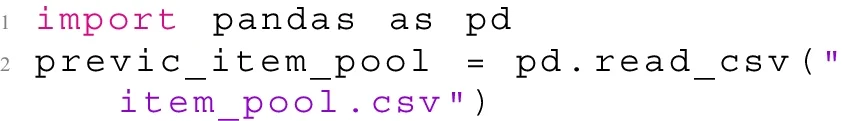

or conda using

**Figure:**
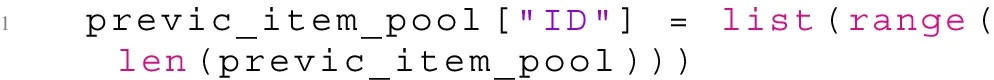

Next, we import the item pool. The item pool stems from the study by Bohn et al. (2025) and can be directly imported from a website from a CSV file^3^. After the file has been successfully downloaded, it can be easily loaded into memory using the pandas (The pandas development team, 2020) library and its read_csv function.

**Figure:**
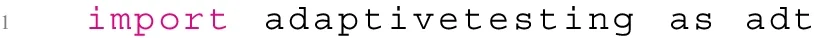

Calling previc_item_pool.head() gives the user an overview of the item pool’s structure. It consists of four columns containing the word itself, the item difficulty, and some residuals from the item parameter estimation. Table 2 shows the first five items in the item pool.

To make the test items easily identifiable later, we add a new ID column where each item is assigned an index depending on its position in the data frame.

**Figure:**
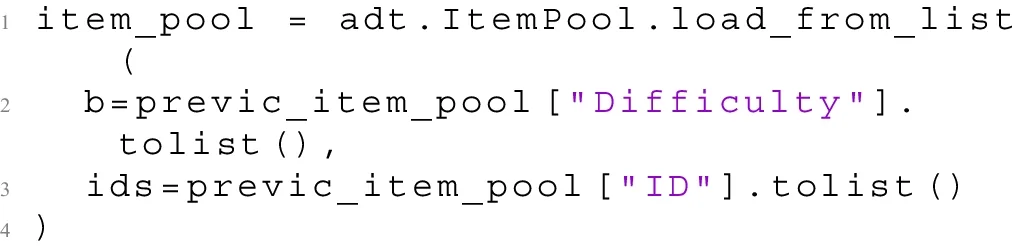

Then, we can continue by importing the package itself. For readability, the alias adt will be used, which gives the users easy access to all relevant functions.

**Figure:**
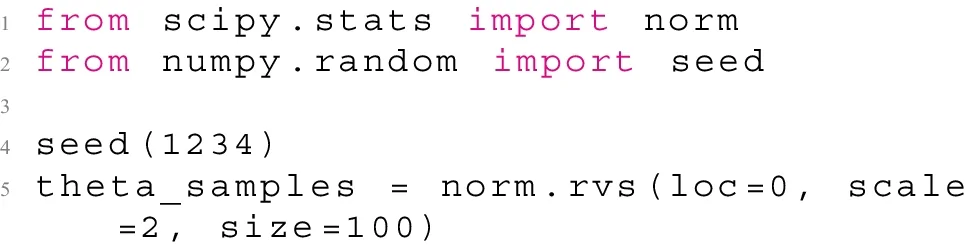

The entire PREVIC item pool is based on the Rasch model (Rasch, 1980), a more restrictive special case of the four-parameter logistic (4PL) model. In the Rasch model, items differ only in their difficulty parameters. Consequently, it suffices to extract only these difficulties and convert them into a format compatible with the software package. The static load_from_list function of the ItemPool class is called, and the Difficulty column as parameter b is passed to the function in order to create the item pool object. For parameter reference, see Eq. 1.

**Figure:**
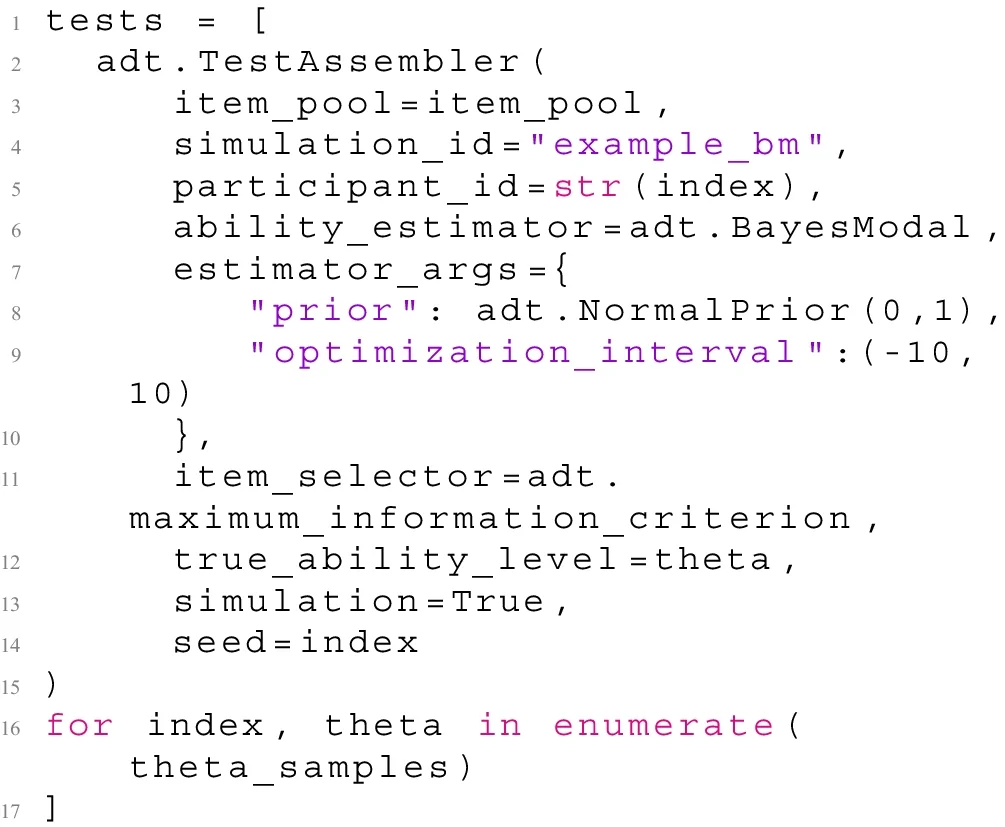

All other parameters are set to their default value, guaranteeing that the 4PL model is correctly restricted to a Rasch model. This item pool object can then later be used to either simulate adaptive tests or adaptively assess a participant.

#### Step 2: Set up simulation parameters and configure adaptive tests

Researchers are often interested in the statistical performance of an adaptive testing procedure and therefore conduct Monte Carlo simulations, which can be easily done using adaptivetesting.

For simplicity, we assume that ability θ in the target population follows a normal distribution, *N*(0, 2), with a standard deviation of 2. Given the standard deviation of the item difficulties in the example above, this assumption appears justifiable.

From this ability distribution, we draw n=100 samples (or observations) using the scipy package.

**Figure:**
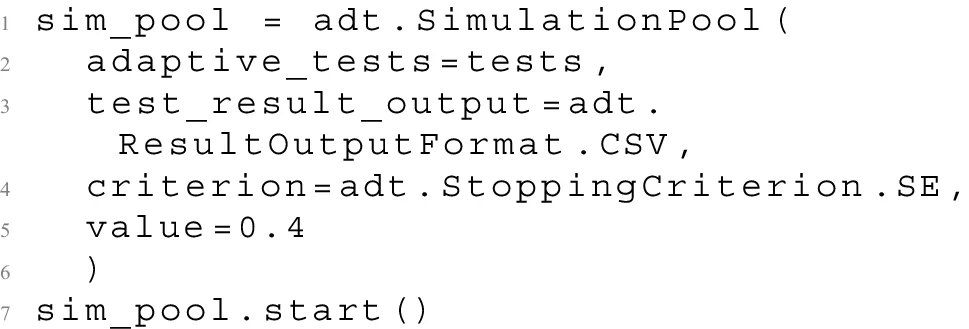

Next, we specify the configuration of the adaptive test to be simulated. This involves selecting both an ability estimation method and an item selection strategy. In this example, we will use BM with a normal prior, *N*(0, 1), for the ability estimation, with the optimization interval set to [-10,10]. For item selection, we apply the MFI criterion.

This setup can be readily implemented using the TestAssembler class. By leveraging Python’s list comprehension, we iterate over the sampled true ability values and instantiate a corresponding test object for each. The expression for index, theta in enumerate(theta_samples) iterates th-rough all previously drawn abilities and returns the value and its index for every draw. To instantiate a test object, we have to pass an item pool to the constructor as well as a simulation ID and participant ID. That way, we can correctly identify the corresponding test results. In order to select BM as an ability estimator, we have to set ability_estimator to the BayesModal class. The behavior of the ability estimator can be further customized using the estimator_args parameter. With this parameter, we can set the desired prior as well as the optimization interval. The MFI is set as an item selector by passing the maximum_information_criterion function to the item_selector parameter. To indicate that the tests are part of a simulation study, the simulation parameter is set to True and the theta value of the current iteration is assigned to the true_ability_level parameter. This way, the true ability value is saved together with the test results to facilitate comparison and further analysis after the simulation. In the context of CAT simulations, it is required to provide responses for every simulated individual and item. With the package, response patterns can either be explicitly defined or generated in the background. For this example, we will let the package automatically generate responses in the background. To ensure our results are replicable, we can set a seed. In this example, we simply choose the simulation index as the seed.

**Figure:**
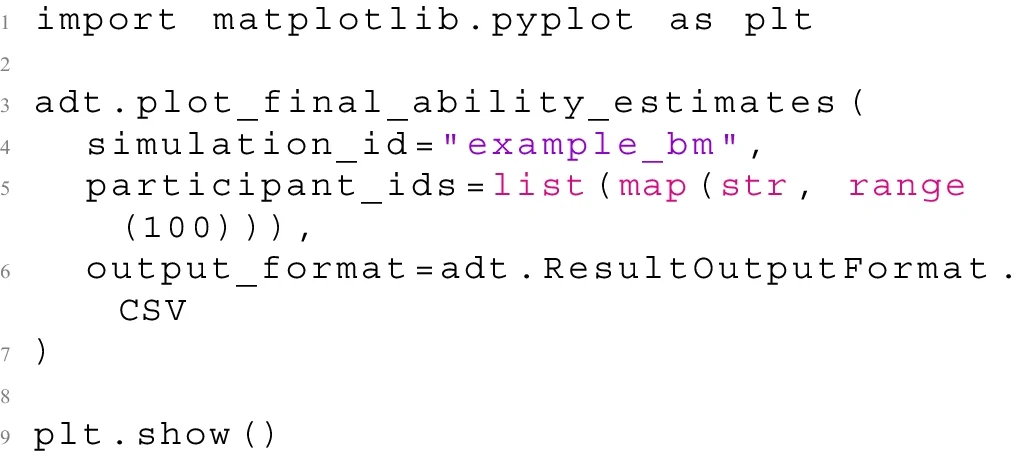

With all test objects created, the simulations can be executed in parallel using the SimulationPool class. The tests objected is passed to the constructor, and the test_result_ouput parameter is set to ResultOutputFormat.CSV to save the test results as a CSV file after the simulation.

In this example, each test should terminate once a standard error of 0.4 is reached. This can be achieved by setting criterion to StoppingCriterion.SE and value to 0.4. Calling the start method of the simulation pool starts the simulations.

**Figure:**
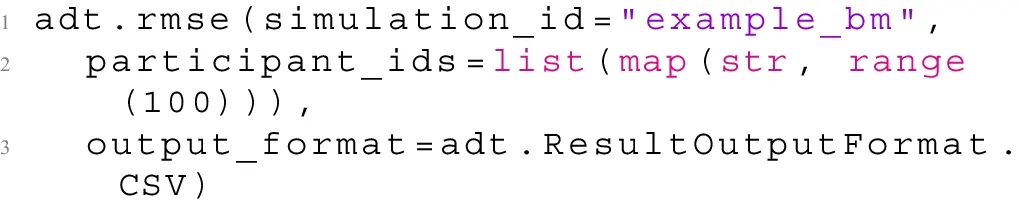

#### Step 3: Analyzing the simulation results

**Fig. 3:**
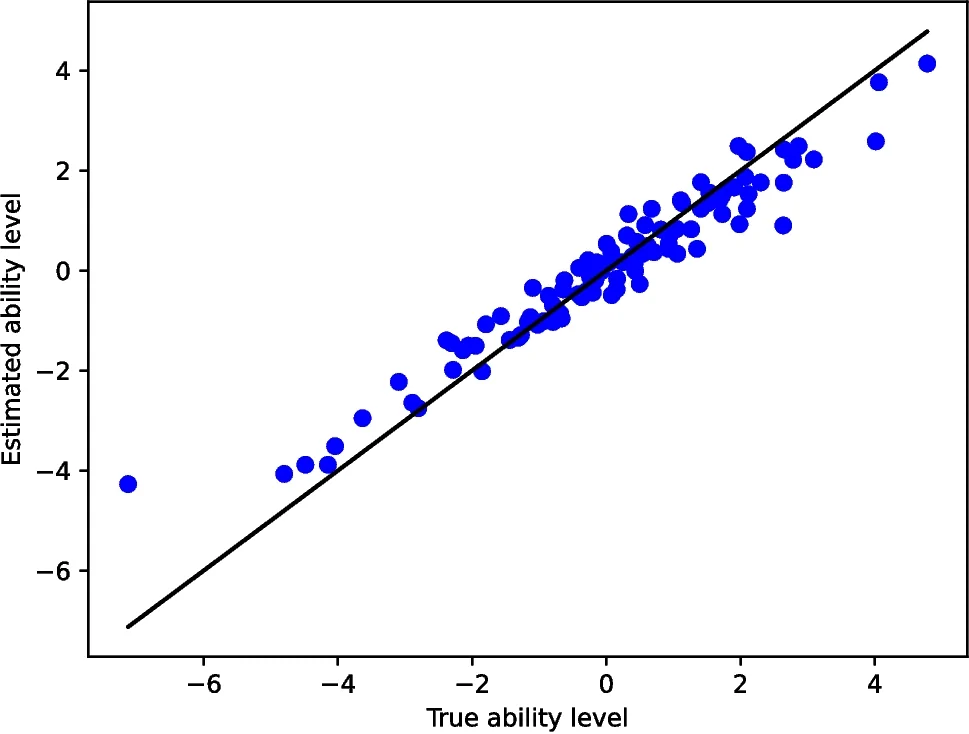
Final estimation result of the adaptive test $\hat{\theta }$ and the true ability values θ using BM as estimator

The simulation results can be easily loaded and analyzed using built-in package functions. For illustrative purposes, we will take a look at the final ability estimates and the estimation trace of a single participant. The final ability estimates can be easily visualized using the plot_final_ability_estimates function, which returns a matplotlib plot object. For this function to work, it is important that the simulation ID, the participant IDs, and the data format are specified. This allows data analysis to be performed completely independently of the simulation. With the show function, the generated graphic is displayed. It is shown in Fig. 3.

**Figure:**
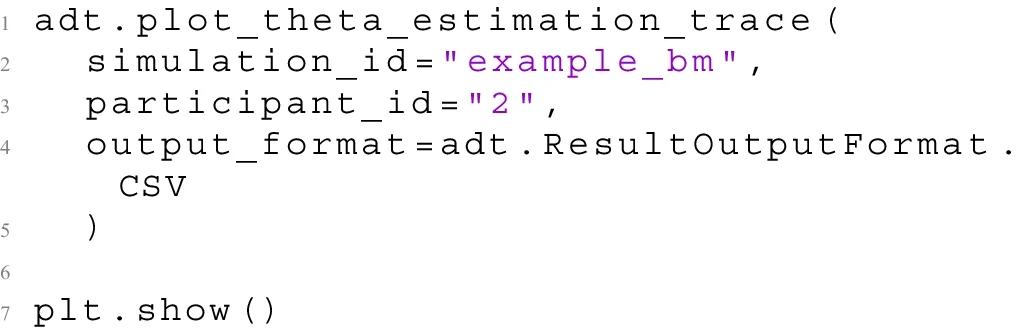

From these results, the RMSE can also be easily calculated using the following expression:

**Figure:**
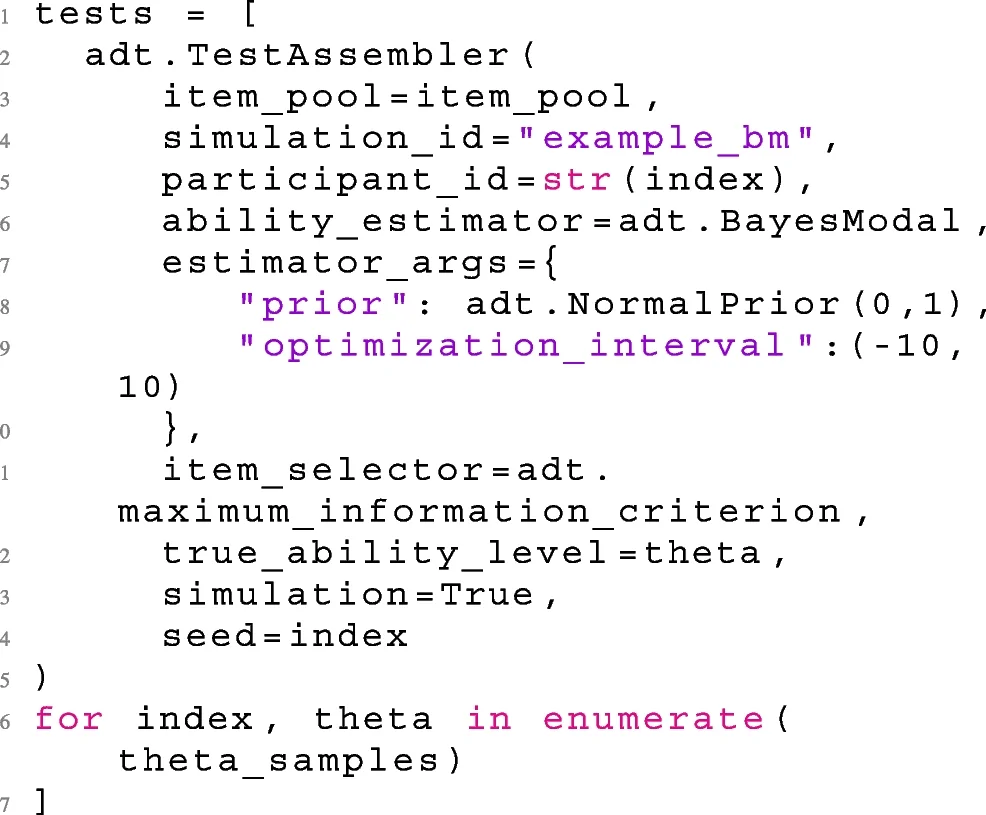

In the case of this simulation, $\text {RMSE}_{\text {BM}} = 0.575$. Figure 3 also reveals that in areas of lower latent ability, there appears to be a tendency to overestimate the ability of the participants. Conversely, in areas of higher latent ability, there seems to be a tendency to underestimate the participants’ ability.

**Fig. 4:**
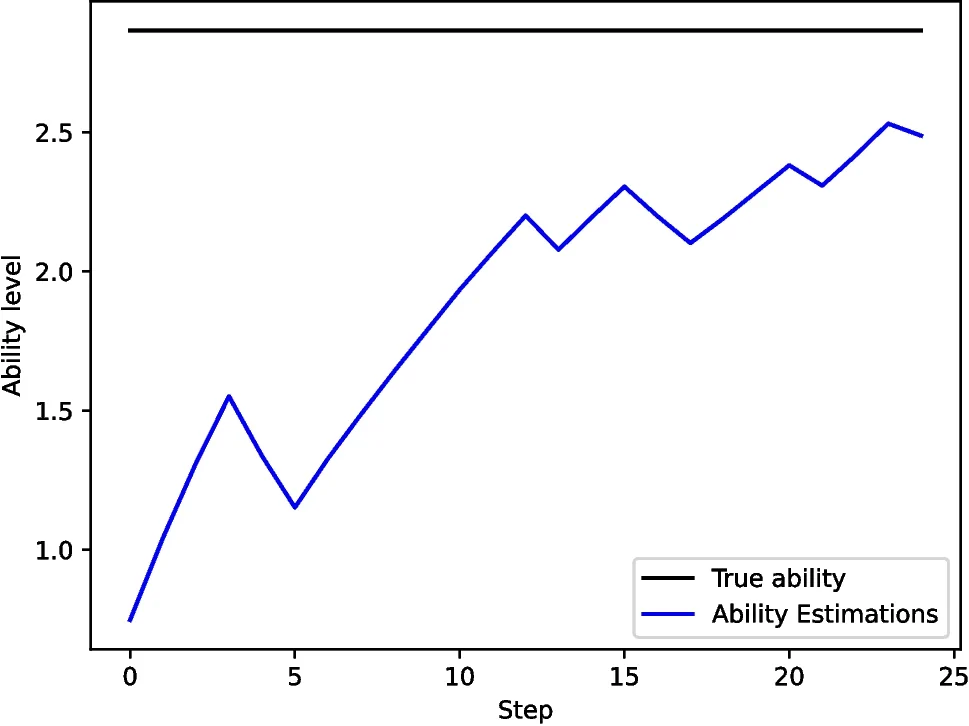
Example ability estimation trace of participant 2

Additionally, users can inspect the estimation trace for specific participants. Using the following function, we can, for example, inspect the estimation trace of participant 2. The resulting plot is shown in Fig. 4.

**Figure:**
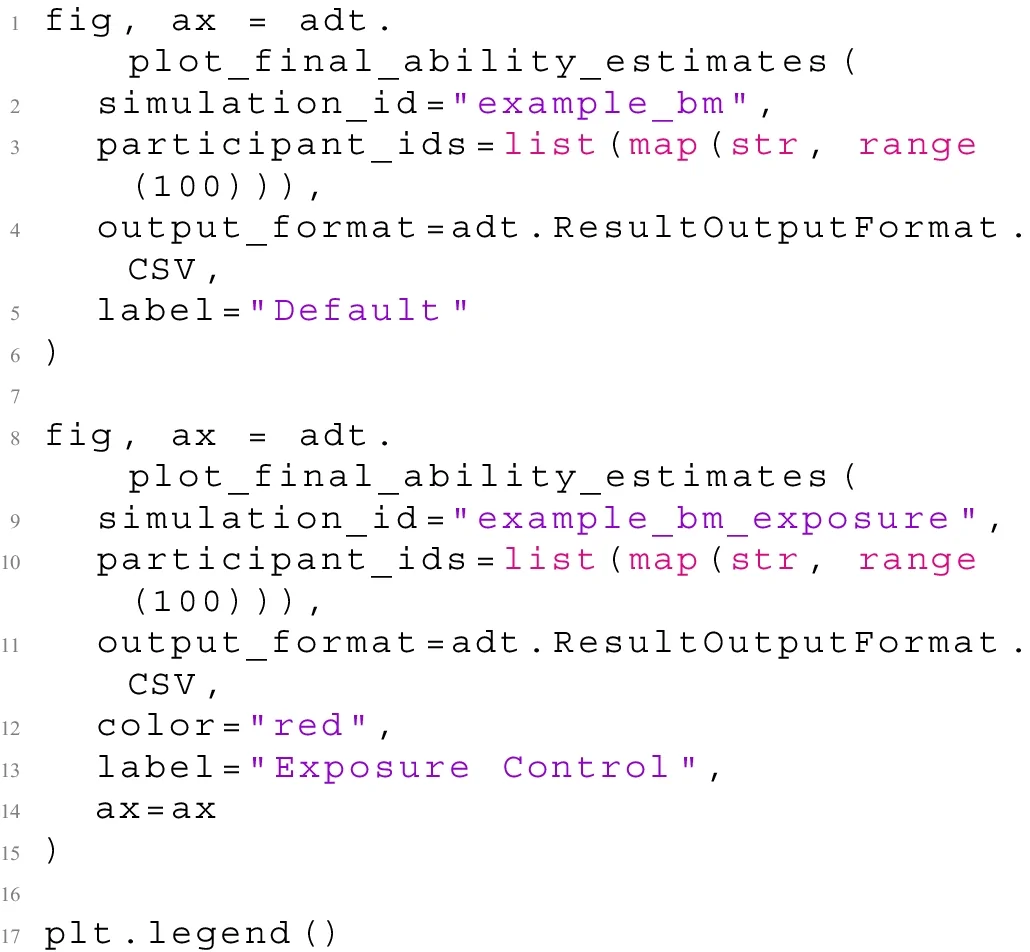

#### Step 4: Modifying the adaptive tests

Of course, the simulation shown earlier implements only a relatively simple adaptive test. For practical applications, the requirements could be more complex. However, even more advanced functionalities can be implemented and evaluated using this package. In the following, we will show how to use randomesque item selection to perform exposure control.

To implement exposure control, we can simply come back to the previous definition command of the adaptive tests and specify the exposure_control and exposure_control_args parameters. For randomesque item selection, the number of items from which the final item is drawn needs to be specified (n_items). For the sake of reproducibility, we assign a seed to each participant – in this case, it is simply the examinee’s index.

**Figure:**
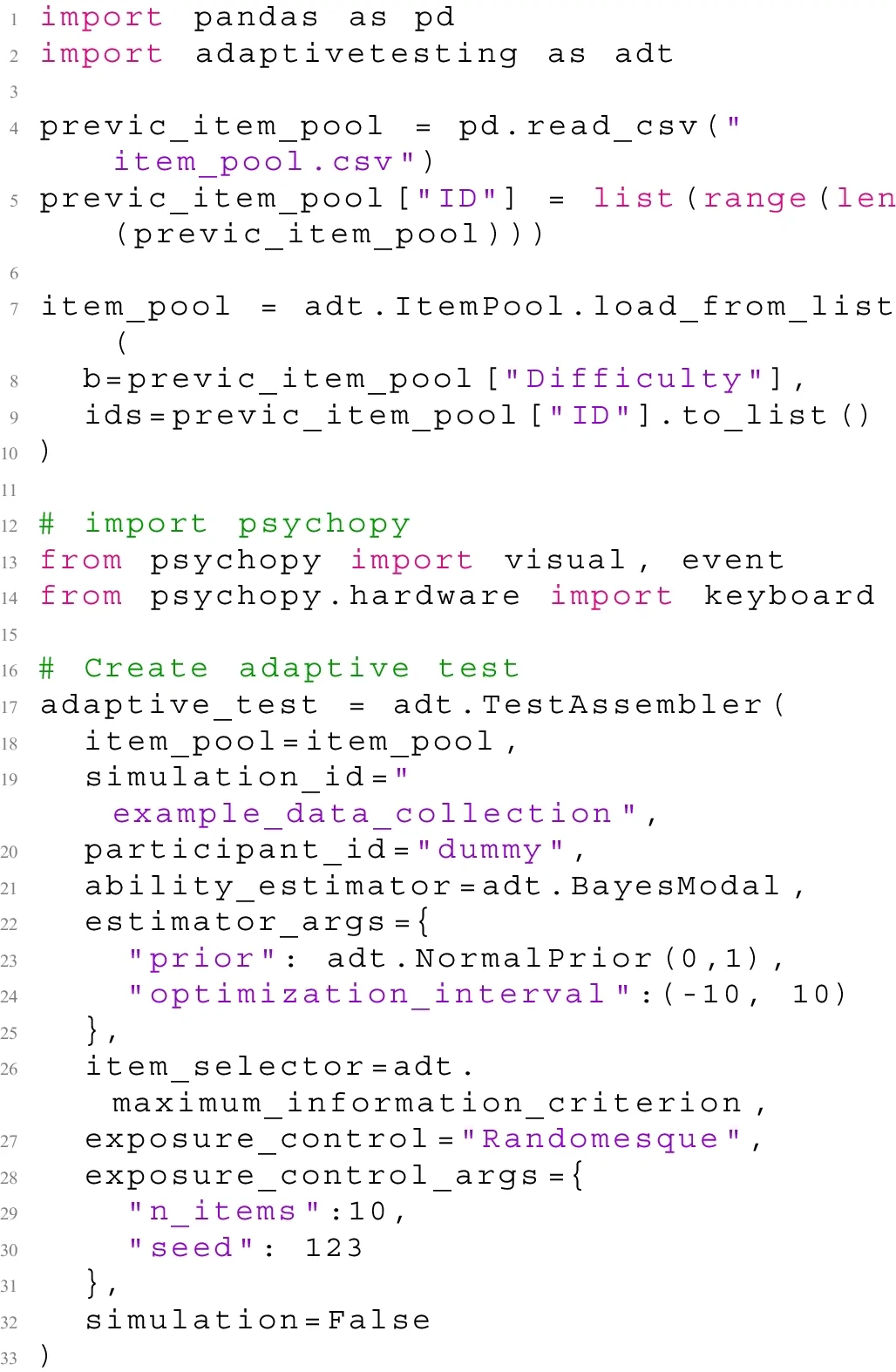

Of course, the tests are then simulated with the previously shown functions.

#### Step 5: Evaluating both simulations

The results of this second simulation can, of course, also be easily analyzed. In addition, it is possible, for example, to plot the final ability estimates from both simulations on the same figure, making it easier to compare the two procedures. To achieve this, we can use the package’s built-in plotting functions, though they will need to be slightly modified so that the results of both simulations are displayed on the same graph.

**Figure:**
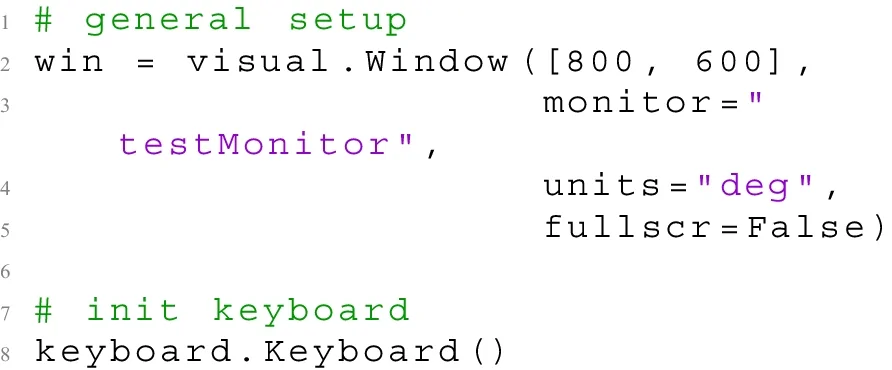

**Fig. 5:**
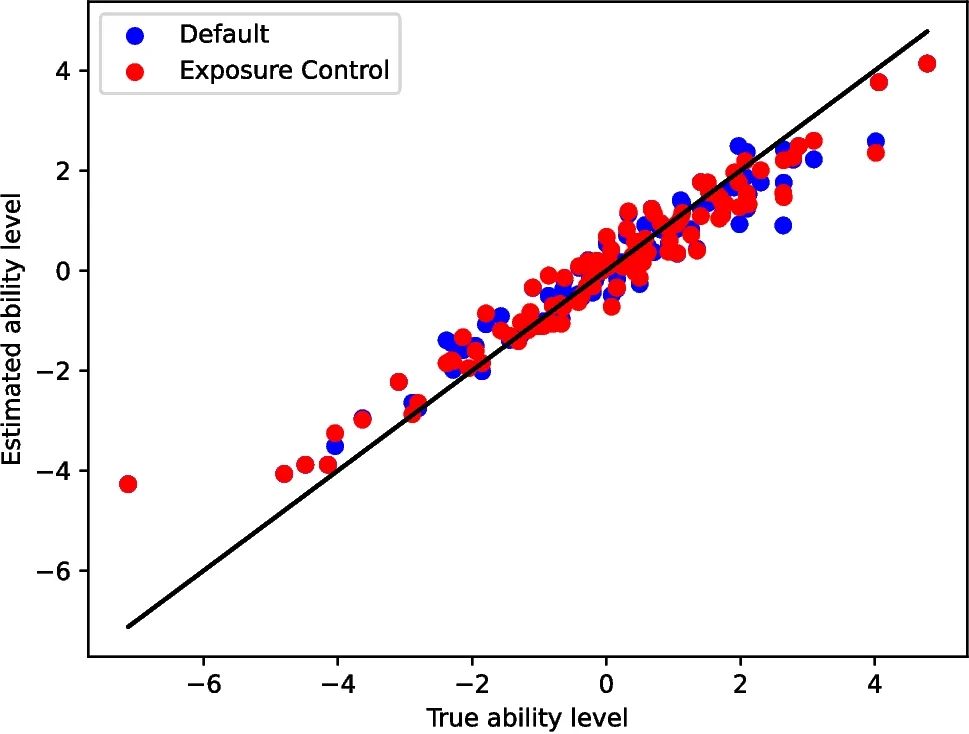
Final estimation result $\hat{\theta }$ and the true ability values θ using BM without (Default) and with randomesque item selection (exposure control)

The created plot is shown in Fig. 5.

It shows that both adaptive procedures yield broadly similar results. The RMSE also indicates that we were able to slightly improve the performance of our tests ($\text {RMSE}_\text {BM} = 0.575$, $\text {RMSE}_\text {ExposureControl} = 0.570$).

### Real-world data collection via PsychoPy

Having evaluated the adaptive test procedure through simulations, we now turn to collecting real data. For this example, we demonstrate how to use PsychoPy (Peirce et al., 2019) in combination with the adaptivetesting package to implement an interactive testing environment.

Currently, the adaptivetesting package does not include a pre-built interface for PsychoPy. This is a deliberate design choice: implementing and maintaining a dedicated interface would significantly limit flexibility and bind the package to a single experiment platform. Instead, the package is designed to interact easily with external experiment software via well-structured APIs.

PsychoPy provides an extensive Python API that enables direct integration with adaptivetesting. However, doing so requires a solid understanding of Python, including dependency management and hardware considerations.

In what follows, we illustrate a minimal working example to show how adaptivetesting can be combined with PsychoPy. For more comprehensive use cases, please refer to the PsychoPy documentation^4^.

#### Step 1: Initialization and test setup

We begin by importing the necessary libraries, loading the item pool, and creating the adaptive test object. This mirrors the earlier simulation setup, with the key difference being that the simulationparameter is set to False. This tells the package to expect real user input rather than simulated responses.

**Figure:**
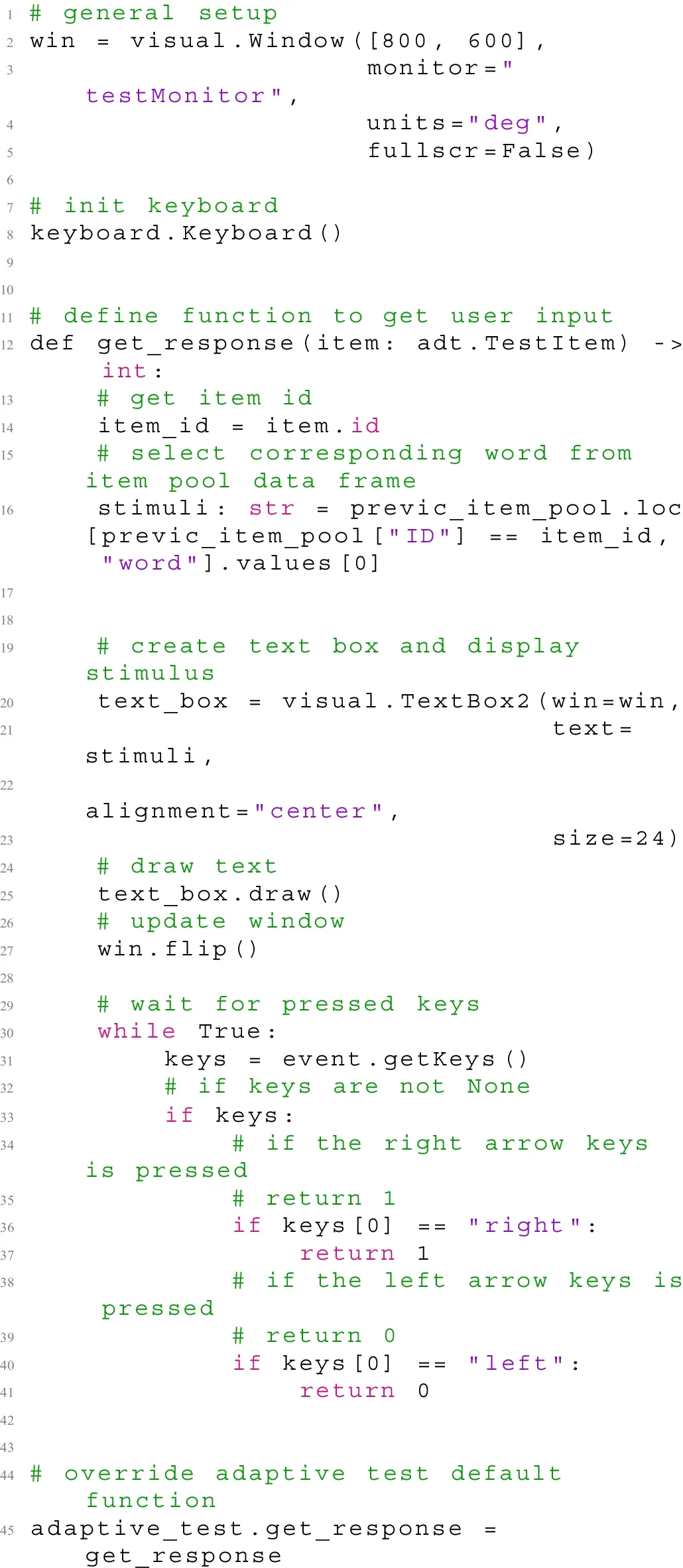

#### Step 2: Display setup and response collection

Next, we initialize the PsychoPy display and keyboard. A window of size 800×600 pixels is created for presenting the stimuli.

**Figure:**
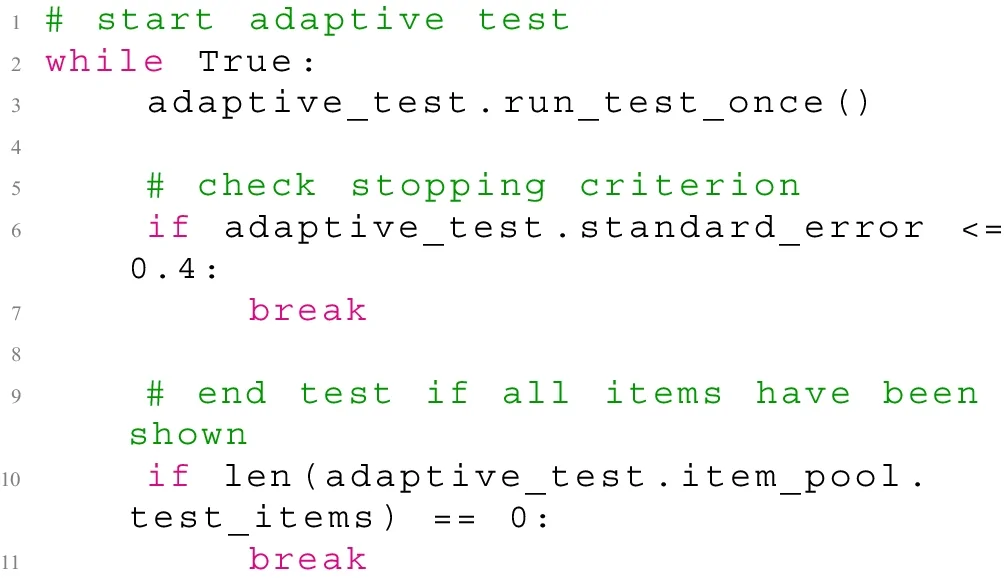

To collect user input, the AdaptiveTest class has a get_response method, which is not implemented by default. Instead, we define a custom get_response function. This function displays the appropriate stimulus (e.g., a word) and waits for a keyboard response. In this example, the user presses the right arrow key if the child can say the word and the left arrow key if not.

**Figure:**
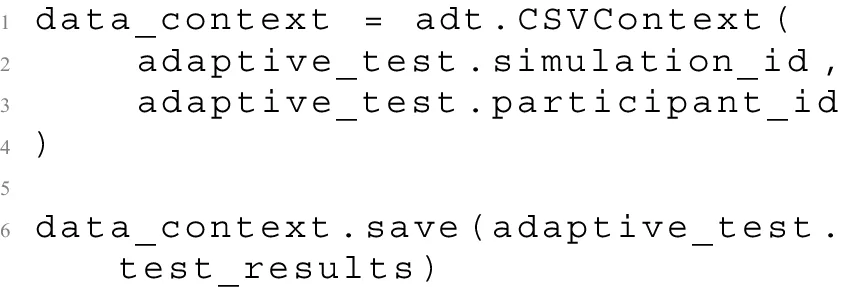

#### Step 3: Running the adaptive test

The adaptive test starts with a while loop that runs the test until a criterion is fulfilled. In this example, the test is stopped if the standard error is ≤0.4 or all items have been shown to the participant. Compared to simulations, the stopping criteria have to be manually checked and implemented. This is because actual applications may be more complex than simulations and require some collection of additional information or another test. This way, one could also run several adaptive tests in parallel. An interface, such as for the simulations, would be an unnecessary restriction on the application of the package.

**Figure:**
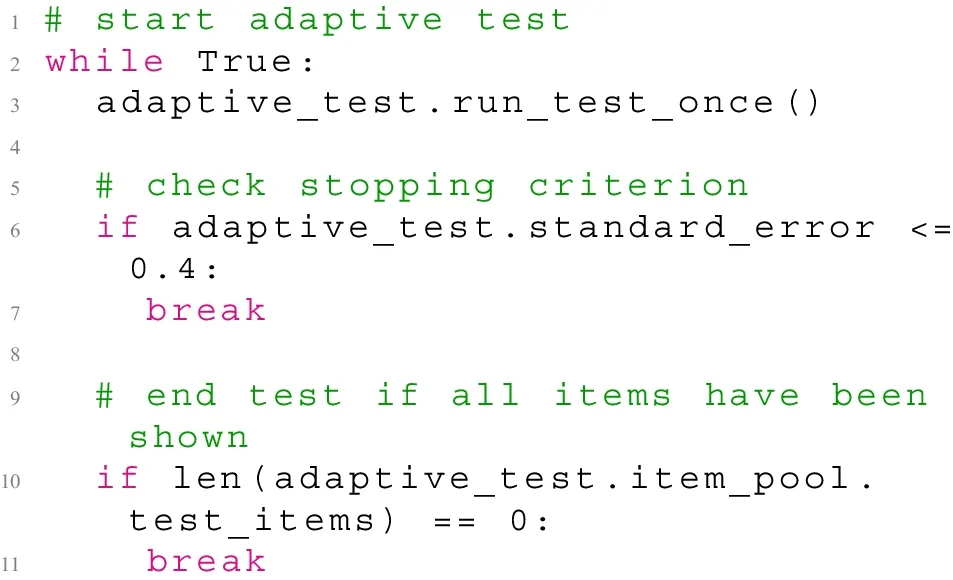

#### Step 4: Saving the results

Finally, the test results are saved to a CSV file using the CSVContext class, resulting in the same directory structure and file naming convention as when simulating adaptive tests.

**Figure:**
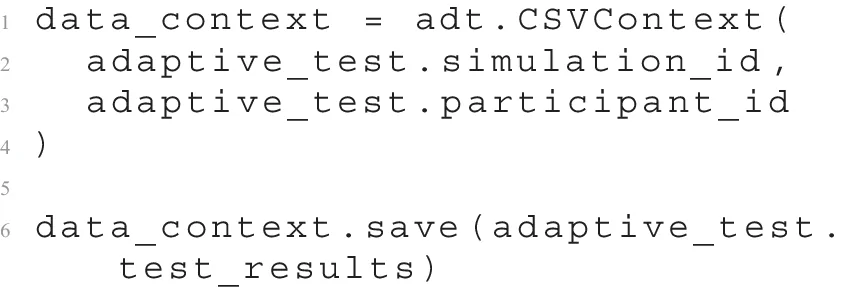

The resulting file can be analyzed in the same way as simulation output, making this approach a seamless extension from simulation to live data collection.

## Discussion

This paper introduced the Python package adaptivetes-ting, which aims to facilitate the development and application of adaptive tests. The package places a specific emphasis on Bayesian methods for ability estimation. It offers an intuitive and easy-to-use interface for both beginners and experienced Python users, while maintaining the flexibility required to accommodate the specific needs of researchers and applicants.

adaptivetesting fills a significant gap in the Python statistical software ecosystem. Existing Python packages for adaptive testing offer only limited functionality and are only suitable for narrow use cases. While R-based alternatives, such as catR, provide a broader range of features, they often lack customizability and flexibility, particularly regarding Bayesian methods for ability estimation.

Importantly, adaptivetesting supports a wide range of the CAT workflow, from test simulation to real-world application. Its modular design requires only minimal code changes to transition from simulated responses to real data collection. Moreover, because the package exposes an API for capturing participants’ responses, it can be integrated with any experimental software that can interface with that API. This enables the use of a wide variety of stimuli and response modalities, including text, images, keyboard input, and mouse clicks.

Although the package includes a range of built-in features, it currently implements only a subset of methods that could potentially be used in CAT. One current drawback is that adaptivetesting does not support a fully Bayesian approach to CAT, which would incorporate not only the uncertainty of the ability estimates but also the item parameters. This is clearly beneficial, particularly as it allows for ongoing updates. A fully Bayesian approach is particularly recommended when a large calibration sample cannot be used (Fink et al., 2025). The benefits of this fully Bayesian approach are clearly evident. Yet, the primary goal of the package’s development was the extension of Bayesian ability estimation. Also, we aimed to create a package that prioritizes speed and practicality over a fully Bayesian approach. However, in the future, the package can be expanded to this end.

Concerning Bayesian ability estimation, the package currently supports several predefined prior distributions. Additionally, users may define custom priors using external libraries such as scipy or by implementing their own functions. It is important to emphasize that users are responsible for ensuring that their configurations are both computationally feasible and diagnostically meaningful. Developing new components may also require a more profound understanding of statistical modeling and numerical methods than using the pre-implemented options.

It is also important to highlight that adaptivetesting currently only supports a selection of models for polytomous response variables, as well as methods for exposure control and content balancing. Techniques, such as shadow testing (Van Der Linden & Veldkamp, 2004), are not available but are planned to be supported with upcoming updates. Moreover, we have to acknowledge that the package currently lacks features for test maintenance, such as drift checks.

Given the rapid current developments and wide array of possible configurations in adaptive testing, the package is designed to be extensible, allowing users to implement alternative algorithms or customize functionality as needed. This extensibility ensures that the package remains useful even as methodological needs evolve.

The adaptivetesting package can also be used together with the R programming language through the reticulate package. Thus, adaptivetesting can also be implemented into existing R-based workflows. However, caution is advised when leveraging the reticulate interface, as unexpected behavior cannot be entirely ruled out.

The example presented in this paper, along with prior research, highlights the practical relevance of adaptive testing in psychological assessment and research. However, the potential applications of adaptivetesting extend well beyond psychology. The underlying models and methods can be applied in a range of disciplines, allowing for reduced test lengths without compromising diagnostic accuracy.

Future development of the adaptivetesting package aims to address current limitations, such as by implementing support for a fully Bayesian approach, more complex IRT models, and shadow testing, while maintaining its accessibility and flexibility. Additionally, advancements in adaptive testing methodology and Bayesian inference will naturally guide future enhancements of the package.

## Conclusion

In sum, adaptivetesting is a novel and innovative tool, providing a framework to make adaptive tests easily accessible – especially in the scientific Python ecosystem – while also facilitating future research and extensions of adaptive tests.

## Data Availability

The dataset used in the application examples is obtained from Bohn et al. (2025) and is available in the following GitHub repository under the Creative Commons Attribution 4.0 International License (CC BY 4.0): https://github.com/manuelbohn/previc. No changes were made to the original dataset.

## Footnotes

1 https://github.com/condecon/adaptivetesting

2 https://github.com/manuelbohn/previc

3 https://github.com/manuelbohn/previc/blob/main/saves/item_pool.csv

4 https://www.psychopy.org/documentation.html

## Code Availability

The package’s source code as well as the here presented code examples and analyses are available in the following GitHub repository: https://github.com/condecon/adaptivetesting.
